# Improving ADRD Diagnosis in Primary Care: Tailoring Blood-Based Biomarker Education for Low-Literacy Populations

**DOI:** 10.1093/geroni/igaf122.2442

**Published:** 2025-12-31

**Authors:** Ignacia Arteaga, Alissa Sideman, Cecilia Alagappan, Joshua Grill, Allyson Rosen, Freddi Segal-Gidan, Loren Alving, Howie Rosen

**Affiliations:** University of California, San Francisco, San Francisco, California, United States; University of California, San Francisco, San Francisco, California, United States; University of California, San Francisco, San Francisco, California, United States; University of California, Irvine, Irvine, California, United States; Stanford University, Stanford, California, United States; University of Southern California, Los Angeles, California, United States; California State University, Fresno, Fresno, California, United States; University of California, San Francisco, San Francisco, California, United States

## Abstract

An estimated 50–70% of symptomatic patients with Alzheimer’s Disease and Related Dementias (ADRD) go unrecognized or are misdiagnosed in primary care, delaying access to supportive services, financial and residential planning, clinical trials, and potentially disease-modifying therapies. One key barrier to timely diagnosis is low educational attainment (e.g., not finishing high school), which is associated with both increased ADRD risk and higher rates of comorbidities. Blood-based biomarkers (BBMs) offer a promising, less invasive, and cost-effective tool to improve diagnosis in primary care. However, individuals with low educational attainment are underrepresented in BBM validation studies, raising concerns about their applicability to diverse populations. ADDPCP, an NIA-funded study, evaluates the accuracy and impact of ADRD diagnosis in primary care across California using clinical tools and BBMs. This presentation reviews the development and validation of educational materials to support BBM decision-making for low-literacy populations. Informed by prior PET amyloid disclosure protocols, we co-developed multi-modal pre-and post-test counseling materials using evidence-based health communication strategies and the Lexile Framework for Reading. These materials—narrated presentations and take-home brochures with pictorial aids—explain the purpose of BBM testing, the risks and benefits of learning results, and the limitations of the information provided. We are in the process of assessing their clarity and relevance through cognitive interviews with 12 matched participants naïve to the study.

